# Pathological and Surgical Observations on Diseases of the Joints

**Published:** 1834-10-01

**Authors:** 


### Pathological and Surgical Observations on the Diseases of the Joints. By *B. C. Brodie*, V.P.R.S. &c. &c. &c. Third Edition, with Alterations and Additions. Octavo, pp. 344. London, 1834.

[Second and concluding article.]

In the last number of this Journal we brought down our analysis of Mr. Brodie's admirable work to the subject of caries of the spine, on which we did not actually enter. It remains for us to place before our readers a digest of the remaining portion of the volume, consisting of about one hundred pages. Mr. Brodie treats consecutively of caries of the spine—tumors and loose cartilages in the cavities of the joints—malignant diseases of the joints—some other diseases of the joints—and, of inflammation of the bursæ mucosæ.

### On Caries of the Spine.

It is scarcely necessary to observe that Mr. Brodie commences his account of a disease, with an enumeration of the pathological changes of which it is made up. He remarks, with reference to the present subject, that opportunities of examining the morbid appearances in a very early stage of disease in the spine are of very rare occurrence. It is evident, however, when the structure of the joints between the bodies of the vertebræ is considered, that they cannot be liable to any diseases, that bear a resemblance to the affections of the synovial membrane which occur in other articulations. But analogy might lead us to expect, what Mr. Brodie's observations have proved to be really the fact, that as two important elements of all joints—cartilage and bone—are present in the spinal column, disease might commence in the one or in the other.

Mr. Brodie has found, in some instances, the intervertebral cartilages in a state of ulceration, while the bones were either in a perfectly healthy state, or merely affected with chronic inflammation, without having lost their natural texture and hardness; but in others it has been manifest that the original disease has been that peculiar scrofulous condition of the bones, the effects of which in the bones and joints of the extremities have been described at length in the chapter with which our notice was concluded in the last number.

Mr. Brodie relates six cases, displaying the morbid alterations, of which the sum gives the history of the disease. He then presents a summary recapitulation of the facts, to which we shall direct our attention first.

In some cases caries of the spine has its origin in that peculiar alteration of the bone, described by Mr. Brodie under the term of scrofulous disease of the joint. Ulceration, in these circumstances, may begin on any part of the surface, or even in the centre of the bone, but in general the first effects of it are perceptible where the intervertebral cartilage is connected with it, and in the intervertebral cartilage itself.

"In other cases the vertebræ retain their natural texture and hardness, and the first indication of the disease is ulceration of one or more of the intervertebral cartilages, and of the surfaces of bone with which they are connected.

There is still another order of cases, but these are of more rare occurrence, in which the bodies of the vertebræ are affected with chronic inflammation, of which ulceration of the intervertebral cartilages is the consequence.

In whichever of these ways the disease begins, if not checked in its progress, it proceeds to the destruction of the bodies of the vertebræ and intervertebral cartilages, leaving the posterior parts of the vertebræ unaffected by it; the necessary consequence of which is, an incurvation of the spine forward, and a projection of the spinous processes posteriorly.

At this period of the disease the membranes of the spinal chord sometimes become affected with a chronic inflammation, which may extend even to the spinal chord itself; and where there is much incurvation, the latter not only becomes incurvated with it, but actually compressed in such a manner as cannot fail to interfere with the due performance of its functions.

Suppuration sometimes takes place at a very early period; at other times, not until the disease has made considerable progress. The soft parts in the neighbourhood of the abscess become thickened and consolidated, forming a thick capsule, in which the abscess is sometimes retained for several successive years, but from which it ultimately makes its way to the surface, presenting itself in one or another situation, according to circumstances.

In the advanced stage of the disease, new bone is often deposited in irregular masses on the surface of the bodies of the neighbouring vertebræ, and where recovery takes place, the carious surface of the vertebra above coming in contact with that of the vertebra below, they became united with each other, at first, by soft substance, afterwards by bony anchylosis. The disposition to anchylosis is not the same under all circumstances; it is much less where the bones are affected by scrofula than where they retain their natural texture and hardness; and this explains wherefore, in the former class of cases, a cure is effected with more difficulty than in the latter.

Occasionally, portions of the ulcerated or carious bone lose their vitality, and, having become detached, are found lying loose in the cavity of the abscess. It is scarcely necessary to add, that the existence of such exfoliations is of itself almost sufficient to preclude all chance of the patient's recovery." 245.

Mr. Brodie remarks in continuation, that caries of the spine may originate in what may be considered as causes from without. It may be produced, for instance, by the pressure of an abscess of the neighbouring soft parts—of an aneurism of the aorta—of any kind of tumor. In such cases, the intervertebral cartilages are in general found to be little, if at all affected, and they project, perhaps of their natural size, while the bones themselves are in a great degree consumed. Where the spine is thus rendered carious consecutively, the symptoms are usually obscure, and the spinal disease is for the most part unsuspected.

Not unfrequently, caries of the spine itself is complicated with caries from external pressure. For example, disease of the vertebræ, or intervertebral cartilages, occasions caries, and this is followed by the formation of abscess. The matter having become accumulated in considerable quantity, the abscess occupies a large space; and by its pressure on the surfaces of the vertebræ in the neighbourhood, causes an extensive caries of them far beyond the boundaries of the original disease.

We shall now select from the cases which Mr. Brodie relates, such particulars as support the preceding observations.

*Case* 1. (LXII.) A girl, eight years of age, died in the year 1808, in the infirmary of the parish of St. George, Hanover Square. She had been an in-patient on account of disease of the spine, the spinous processes of the dorsal vertebræ forming a preternatural projection posteriorly. Abscesses had also burst in the groin.

On examination of the body, there was found at the posterior part of the abdomen a confused mass of soft substance, which proved to be the parietes of an abscess communicating with the orifices in the groin. The bodies of the lowest dorsal and three superior lumbar vertebræ were found at the posterior part of the abscess, nearly consumed by caries. There were no remains of the intervertebral cartilages between the tenth and eleventh dorsal, nor of those between the third and fourth lumbar vertebræ. These intervertebral spaces were filled with pus, and the opposite surfaces of the vertebræ were carious, but only to a small extent. The central part of the intervertebral cartilage between the ninth and tenth dorsal vertebræ had been completely absorbed, and pus was found in its place. Externally to this, the concentric layers of elastic cartilage were entire, though somewhat altered from their natural appearance.

In the next case, (Case LXIII.) besides extensive caries of the dorsal vertebræ and ulceration of the inter-vertebral cartilages, the latter intervening between the third and fourth, fifth and sixth, seventh and eighth, tenth and eleventh dorsal vertebræ, and also that between the eleventh dorsal and first lumbar vertebræ were found in a perfectly natural state towards their circumference, but of a dark colour in the centre. On the surfaces towards the bones they, as well as the bones themselves, were in a state of incipient ulceration, but without any appearance of pus having been secreted. The bones of the vertebræ had everywhere their natural texture and hardness.

*Case* 3. (XLV.) A girl, aged 19, was admitted into St. George's Hospital, on the 30th May, 1821, with violent pain in the left leg, from the knee to the foot. Previously to her admission she had suffered from pain in the loins, which was relieved by cupping. Soon after her admission this symptom returned, accompanied with a tumor in the loins on the right side. Sixteen ounces of matter were discharged from this by puncture, hectic symptoms were established, and on the 3d of August she died.

On dissection, the bodies of the three or four inferior lumbar vertebræ were found preternaturally vascular, and of a dark, and almost black colour; but they retained their natural texture and hardness, and had undergone none of those changes which mark the existence of the scrofulous affection of the bones. The intervertebral cartilages were in a natural state; but the body of one of the vertebræ was superficially ulcerated for about the extent of a sixpence on one side, towards the posterior part. A large abscess communicated with this ulceration, and occupied the situation of the psoas muscle of the left side, extending downwards to the groin.

*Case* 4. (LXVI.) A man, aged 45, was admitted into the hospital with an abscess in the left groin. On being confined to the horizontal posture this disappeared, and then presented itself over the left os innominatum. Forty ounces of pus were discharged from this by puncture. The patient died exhausted by hectic.

On dissection, it was found that the cancellous structure of all the dorsal and lumbar vertebræ was of a dark red colour, and softer than natural, so that they might be cut with a common scalpel, or even crushed by the pressure of the thumb and fingers.

The opposite surfaces of the bodies of the second and third lumbar vertebræ, and of the cartilage between them, at the posterior part, were extensively destroyed by ulceration. Anteriorly, the bones and the intervertebral cartilage were entire, and the latter was in a perfectly natural state; but the bones throughout were of a dark and almost black colour.

On one side of the body of the twelfth dorsal vertebra, there was a small ulcerated spot, forming an opening which extended itself into a small cavity in the centre of the bone. This bone was also of a black colour ; but the intervertebral cartilages belonging to it, as well as the intervertebral cartilages connected with the other vertebræ, were in a perfectly natural state.

These cases appear to be sufficient to display the principal pathological features of caries of the spine. On the one hand, they seem to shew that the intervertebral cartilage may constitute the primary seat of the disease; on the other, it is observed to be limited to the bone. It may be noticed that, in one case, the vertebræ were softened, as in scrofulous disease of the bones of the extremities. In two other cases related by Mr. Brodie, caries of the spine was co-existent with tubercles in the lungs, and with other marks of the strumous constitution. In one of these instances, the vertebræ retained their natural hardness, but appeared to be less vascular than natural; in the other, the bodies of the vertebræ were softened, and the cancellated structure of the ribs was filled with a curdly matter.

We may now proceed to the consideration of the symptoms of caries of the spine.

Mr. Brodie regrets that he is unable to offer diagnostic marks of a satisfactory description, between ulceration of the intervertebral cartilage and caries of the body of the vertebra. As caries of the latter is the common consequence of both, it might have been anticipated that a certain similarity would attend their symptoms. This anticipation appears to be borne out by facts. Mr. Brodie, however, hints his suspicion, that the following considerations may in some degree enable us to determine, or rather to suppose, the actual nature of the case.

"I suspect that, where the disease is of scrofulous origin, affecting the cancellous structure of the bones, it is more immediately followed by suppuration, than where it commences in the form of ulceration of the intervertebral cartilages ; and that in cases of the latter description the pain and tenderness in the situation of the carious portion of the spine is more considerable than in those of the former." 247.

It must consequently be understood that, when Mr. Brodie speaks of the symptoms of caries of the spine, his observations are intended to be applicable to the various cases of this description, excepting only those in which the alteration is a secondary result of the pressure of a tumor in the neighbourhood.

Caries of the spine is most frequent in those whose constitutions were originally weak, or whose bodily powers are in any way impaired. Sometimes the patient has previously enjoyed good health.

Mr. Brodie remarks that two orders of symptoms may be the results of this disease, independently of the effects which, in its most advanced stage, it produces on the general system. The symptoms in question are, first, those which are the immediate consequence of the morbid condition of the vertebræ themselves, and of the intervertebral cartilages; and, secondly, those which arise from pressure on the spinal cord, or from irritation propagated to it, or to the nerves arising from it.

The symptoms of the first class are briefly enumerated by Mr. Brodie.

"1st. Pain and tenderness in the situation of the carious vertebræ.

2dly. Curvature of the spine forward, with an angular projection of the spinous processes posteriorly, the result of the bodies of the vertebræ having been destroyed, while the other parts of these bones remain entire.

3dly. Abscess commencing imperceptibly, but at last presenting itself as an external tumour." 249.

The second order of symptoms may be stated thus.

"4thly. Pains, loss of sensation, coldness, muscular spasms, and paralysis of the extremities.

5thly. Derangement of the functions of the various viscera, which are capable of being influenced by that portion of the spinal chord which is implicated in the disease." 249.

To the observations which succeed, we entreat the active attention of our readers. Indeed, the whole chapter on caries of the spine is a happy illustration of Mr. Brodie's method and style. The essence of a long experience and of patient observation is filtered through a close analytical process, and presented in the most concentrated form that the operations of a philosophical mind can effect. Were there many such chapters in many such works, the satire of D'Alembert on authors would be idle,* and the task of the critic would be vain. The latter could condense little, and condemn not at all, and, like Ramsay's shepherd, he must—

Break his pipe, and never whistle mair.

To return to the symptoms of caries of the spine. Mr. Brodie remarks that the whole of those enumerated above are not observed in every instance, nor do those which exist always shew themselves in the same order. They are modified and altered by various circumstances to a great extent, and few diseases display, in individual instances, such diversities of form, or require in the early stages more experience and discrimination, to enable the surgeon to form a correct diagnosis.

Mr. Brodie first considers the symptoms of caries, which are common to the affection in different portions of the spinal column. He afterwards particularizes those which are peculiar to disease of its individual parts, or, at least, of the three great anatomical divisions.

"In the majority of cases, the first symptom which the patient notices is pain referred to that part of the spine in which the caries exists; at first trifling, but becoming more severe afterwards. The pain is aggravated by any sudden motion of the spine; by percussion, or by a jar communicated to it in any other way; as by stamping on the ground, striking the foot accidentally against a stone, sneezing, or coughing. In the advanced stage of the disease the pain is sometimes so severe, and so easily induced, that the patient cannot bear the slightest movement. Yet, in other cases, there is sometimes no pain in the spine whatever, from the first access of the disease to its termination." 250.

---

* L'auteur se tue à alonger, &c.

Mr. Brodie mentions two facts illustrative of the latter observation. In the case of a young gentleman, in whom the disease had been going on for years, and who displayed, on examination after death, a large abscess connected with caries of four or five of the dorsal vertebræ, no pain had ever been complained of. In another instance, the patient had not experienced any pain for the two or three years preceding his decease, and was supposed to have been cured; but, on dissection after death, Mr. Brodie found the bodies of the vertebræ still carious, and an abscess containing half a pint of matter in connexion with them.

As a general rule, pain precedes the appearance of curvature, for a period which varies from three months to two years, and is sometimes even longer than this. It is referred to the affected part. Where pain in the early stage is wanting, a circumstance which, on the whole, is rare, there is usually some derangement of the general health, weakness of the extremities, or other symptoms, shewing that the patient labours under some kind of disease, without indicating its exact nature and locality.

The distortion of the spine in these cases is peculiar. It forms an angle projecting backwards, an alteration which can only be effected by destruction of the bodies of one or more of the vertebræ. The curvature is in general gradually established. A young woman, however, who had made no previous complaint, experienced, after a slight exertion, a sensation as if something had given way in her back, and immediately afterwards lost the use of her lower limbs. This was followed by an angular projection of the spinous process of one of the inferior dorsal vertebræ, and a large abscess, which presented itself on one side of the abdomen; and the patient ultimately died. In another case, after the curvature had taken place, the form of it appeared to vary, in consequence of the diseased vertebræ admitting of being moved to a certain extent on each other; these motions being attended with increased pain, both in the spine and in the lower extremities. This patient ultimately recovered.

"Curvature of the spine in the direction forwards may arise from other causes, as a weak condition of the muscles, or a rickety affection of the bones. In general, in such cases, the curvature occupies the whole spine, which assumes the form of the segment of a circle. At other times, however, it occupies only a portion of the spine, usually that which is formed by the superior lumbar and inferior dorsal vertebræ; as I have ascertained, not only by examinations during life, but by dissection after death. Here the curvature is always gradual; never angular; and thus it may be distinguished from the curvature arising from caries. Nevertheless, I am satisfied that these different kinds of curvature, arising from different causes, have frequently been confounded with each other; and that some of the cases which have been published as examples of caries of the spine, and in which it may, at first, be a matter of surprise that so complete and so speedy a cure has been effected, have in reality been cases of an entirely different malady."* 253.

Mr. Brodie has already stated that suppuration appears to take place at an earlier period, in cases in which the disease originates in the bone, than in those in which it begins in the intervertebral cartilages. In cases of the

---

* Some excellent observations on this subject have been published by Mr. Earle, in the Edinburgh Medical Journal for January, 1815.

latter kind, the disease will sometimes proceed to a remarkable extent without the formation of abscess, a circumstance extremely fortunate for the patient, as his chances of recovery are greatly increased by it. But an abscess frequently exists, although no external signs of it are present. We frequently find evidence of this on dissection.

Caries of the vertebræ not uncommonly goes on for two or three years, before there are any certain indications of abscess. In one case, in which the disease was in the lumbar vertebræ, an abscess appeared in the groin at the end of eight years; and in a case of disease of the dorsal vertebræ, sixteen years formed the lengthened interval.

The formation of abscess is usually attended with some derangement of the general health—slight fever—loss of flesh—perhaps rigors. These symptoms may at first be relieved in some degree by the bursting of the abscess, but they afterwards recur in an aggravated form—the patient wastes under the influence of hectic—and some kind of fatal visceral disease is established.

Mr. Brodie next considers the peculiar symptoms produced by the affection of one or other part of the vertebral column.

When there is caries of the cervical vertebræ, the patient complains at first of pain in the neck, which is aggravated by every motion of the head, and which is not unfrequently mistaken for the muscular pains and stiffness connected with what is commonly called a stiff neck from cold. The pain gradually increases, and Mr. Brodie has found it more liable to be severe, than when the seat of the disease is in the lower part of the spine. When the spine has become incurvated forward, the angular projection posteriorly is observed to be trifling, except when the seventh cervical vertebra is implicated in the disease; a difference easily explained by the length of the spinous process of that vertebra.

Abscess connected with diseased cervical vertebræ usually presents itself among the muscles on the side of the neck. Occasionally it breaks into the pharynx. Mr. Brodie has seen one case in which it penetrated into the theca vertebralis, and the whole spinal chord was bathed in pus.

"At an early period of the disease, the patient frequently complains of pains in the arms and shoulders. After some time these pains subside, but they are followed by complete paralysis of the upper extremities; while the muscles which derive their nervous influence from the spinal chord below the neck remain subject to the will. In a still more advanced stage of the disease, the paralysis extends to the muscles of the trunk and of the lower extremities. Then there are pains in the abdomen, which becomes distended and tympanitic; the bowels being at the same time obstinately costive. In all cases, there is pain in the occiput and temples; which is, however, most severe when the disease is situated in the two or three superior vertebræ. Not unfrequently the transverse ligament of the second vertebra is destroyed, and the consequence is, a dislocation of the odontoid process. Sometimes the dislocation is complete, and the patient, from the pressure made on the spinal chord, expires as suddenly as if the latter had been divided transversely. More frequently it happens that the displacement of the odontoid process is somewhat restrained by the pressure of the dura mater which lies over it. There is then some degree of pressure on the spinal chord, sufficient to excite irritation, but not sufficient to destroy its functions. Under these circumstances, the patient complains of increased pain in the head, followed by convulsions, stupor, dilated pupils, and other symptoms of effusion of fluid on the brain; and on examining the body, after death, we find that such effusion has actually taken place, there being a collection of fluid in the ventricles, or in the base of the cranium, or in both of these situations." 266.

In cases of caries of the superior dorsal vertebræ, besides the usual pain and tenderness of the affected parts, the patient complains of pain and a sense of constriction in the chest; and when the disease is in the inferior dorsal vertebræ there is a similar sensation in the epigastrium, pain in the abdomen generally, and a disturbed state of the functions of the alimentary canal. Occasionally the urine is alkaline, or contains albumen, from which circumstance, in connexion with the existence of pain in or near the region of the kidney, it is sometimes difficult to determine, in the first instance, whether the patient labours under caries of the spine, or disease of the kidney.

When the spine is incurvated forwards, in consequence of the destruction of the bodies of the dorsal vertebræ, the angular projection behind is more distinct than where the lumbar or cervical vertebræ are affected. This is owing to the length and disposition of the dorsal spinous processes. When the curvature is considerable, the thorax is altered in figure, its diameter from above downwards being diminished, while its other diameters are increased. When the superior dorsal and inferior cervical vertebræ are both implicated in the disease, a large protuberance presents itself between the superior angles of the scapulæ, and the neck appears shortened, as if it had descended or sunk between the shoulders.

As the disease advances, the patient, in some instances, complains of pains, which are referred to one groin and hip. This circumstance not unfrequently occasions an error in diagnosis on the part of even practical surgeons. Afterwards pains, and a sense of constriction, are felt in the legs and thighs. Then the muscles are found to be not properly under the dominion of the will, so that the patient occasionally loses a step, or trips in walking. This is probably followed by a complete loss of voluntary power. In some cases there is an entire paralysis; the muscles of the lower extremities never acting under any circumstances; while in other cases, although they do not act under the influence of volition, they are subject to involuntary contractions or spasms. Occasionally, but rarely, the loss of voluntary power is attended with a total loss of sensibility. Paralysis of the bladder, and incontinence of the urine and fæces, sometimes accompany paralysis of the lower limbs.

"A considerable time generally elapses before abscess connected with caries of the dorsal vertebræ presents itself externally. Sometimes it shews itself on the posterior or lateral, or even on the anterior part of the chest, having penetrated through one of the intervertebral spaces. More commonly it makes its way downwards through the posterior mediastinum, and behind the small muscle of the diaphragm; and then, taking the course of the psoas muscle, passes behind the crural arch, and shews itself in the anterior and upper part of the thigh. It is not uncommon for the abscess to form a large tumour on one side of the abdomen, occupying the whole, or a great part, of the space between the false ribs and the groin, pushing the viscera to the opposite side, and, at last making its way to the surface through the abdominal muscles. But a great length of time may elapse before it reaches this termination. I have known such an abscess to remain neither increasing nor diminishing in size, nor being materially changed in its situation, for several successive years: in some instances being a soft and compressible tumour, in which the fluctuation of matter was distinctly perceptible; in others, appearing like an irregular mass of solid substance, closely attached to the posterior and lateral parts of the spine." 260.

When the lumbar vertebræ are affected with caries, there is usually pain in the region of the loins, which is aggravated by stooping, by suddenly turning round, or by percussion. Sometimes the pain is confined to the vertebræ themselves; at others, it extends to the sides of the abdomen and the crista of the ilium.

When an abscess is formed, it usually either descends in the direction of the psoas muscle, and appears in the upper and anterior part of the thigh—or, passing by the outer edge of the quadratus lumborum and sacro-lumbalis muscles, it shews itself on one side of the loins—or in some rare cases, it takes the course of the spermatic chord, and projects through the abdominal ring, where a superficial surgeon might readily mistake it for a hernia—or, descending into the pelvis, it follows the direction of the great sciatic nerve, and proceeds to the posterior part of the thigh. It may reach this situation in another way. Mr. Brodie has known an abscess which, originating in the loins, had presented itself in the groin, disappear from this part suddenly, and, after an interval, be discovered in the posterior part of the limb, behind the lesser trochanter. In a case of this description, Mr. Brodie ascertained, on examination after death, that the abscess had taken the course of the psoas and iliacus muscles to their insertion, and had afterwards extended backwards over the inferior edge of the quadratus femoris. Mr. Brodie makes the observation, that it is not uncommon for abscess connected with caries of any portion of the spine, to alter its course in this manner. He supposes that this circumstance affords an explanation of some of those cases, in which it has been thought that an abscess has been suddenly removed by absorption.

It very rarely happens that caries of the lumbar vertebræ proceeds to the extent of occasioning a perceptible alteration in the figure of the spine. This is easily accounted for by the anatomical formation of those vertebræ. This last consideration will enable us to understand another peculiarity of caries of the lumbar vertebræ—the absence, in the majority of cases, of pains, muscular spasms, paralysis, and loss of sensibility in the lower limbs. In these cases, the thickness of the bodies of the vertebræ is commonly sufficient to prevent the caries from reaching the theca vertebralis. In one case, in which the patient *did* complain of numbness of the legs and thighs, Mr. Brodie found, on dissection, that the *theca vertebralis* was in no part exposed; but that there was a large abscess on each side, surrounding the origin of the anterior crural and obturator nerves, and thus explaining the diminished sensibility of the parts to which they were distributed.

"In systematic works on surgery, the lumbar or psoas abscess is usually described as if it were (in some instances at least) a specific or primary disease, having its origin in the psoas muscle. But, according to all the experience which I have had in these cases, this is altogether a mistaken view of the subject. I cannot say that such an abscess never takes place in the loins; but I certainly believe that it is of very rare occurrence. In examining cases of lumbar abscess after death, I have always found caries of the vertebræ, in which the abscess has manifestly originated. In general the disease of the vertebræ has been so obvious, that it could not have been overlooked by the most superficial observer; but, in some instances, the real nature of the disease has not been detected until after a careful dissection. In one instance, on examining the body of a patient who died in St. George's Hospital with an extensive suppuration in the loins, the soft parts having been entirely removed, not the smallest appearance of disease presented itself in the lumbar vertebræ, and I conceived that I had at last met with a case of genuine psoas abscess; when, almost accidentally, a small opening was discovered on one side of the spine, in a part which had been covered by one of the attachments of the psoas muscle, just large enough to admit a common probe, and forming a communication between the cavity of the abscess, and one of the intervertebral spaces. On a further dissection, it was ascertained that the intervertebral cartilage had been completely destroyed by ulceration, except at its circumference, and that the opposite surfaces of the bodies of the two contiguous vertebræ were extensively carious." 263.

The consideration of the symptoms of caries of the spine is succeeded by a chapter on its treatment.

This is comparatively obvious and simple. The principal difficulty experienced by the surgeon is in ascertaining the nature of the malady. That determined, the principles which guide his practice are neither obscure nor difficult of application.

It might be supposed that none could be bold enough to call in question the prudence, and few to deny the indispensable necessity of rest in the horizontal posture, and that for a considerable period. Counter-irritation, in the shape of caustic issues, is also a remedy that would readily occur to the mind of the reflecting surgeon. These means, with the administration of the remedies adapted to the general condition of the patient in each individual case, appear to constitute the essential items of the treatment.

From the moment that the nature of the case is ascertained, the patient should be confined to his bed or couch. Where severe pain is experienced in the vertebræ, the patient will readily submit to this discipline; but where the suffering is inconsiderable, the persuasions and the firmness of the surgeon are required to indicate and enforce its paramount necessity. The bedstead contrived by Mr. Earle will be usually most convenient. Where the disease has existed for a length of time, and much angular curvature of the spine has taken place, the patient should rather lie on his side than on his back, or, if this be disagreeable or inconvenient, he should be so supported by cushions and pillows that the position in which he is may not tend to restore the spine to its original figure. Mr. Brodie adds an earnest and important caution to those who are inclined to meddle with diseased spines, and attempt or affect to remove the displacement by mechanical means, or by manipulation. It is scarcely necessary to expose the ignorant and mischievous absurdity of such persons.

When the disease is situated in the dorsal or the lumbar vertebræ, the patient may be provided with a bandage, including some stripes of whalebone, and somewhat resembling the stays worn by females, but extending as low as the symphysis of the pubes, the os sacrum, and the great trochanter, and as high as the neck. This will operate like splints, fixing the pelvis and thorax in the same relative position. A less efficient support may be given to the cervical vertebræ by means of a cushion adapted to the shape of the lateral and posterior parts of the neck, and extending from the upper part of the back to the occiput.

Mr. Brodie concurs with those who advocate the use, and maintain the advantages of caustic issues. He acknowledges, at the same time, that some cases occur, in which they appear to be productive of little or of no utility. He seems disposed to think that in diseases of the spine, as in those of the joints, issues are of most benefit where the intervertebral cartilages are affected, and of least advantage where the vertebræ are affected with scrofulous disease. We need scarcely add that the former cases are those in which there is most pain. Mr. Brodie is also of opinion, that issues are applicable to the early stage of caries, and are valueless after suppuration has occurred.

The duration of the treatment is an important consideration. It is difficult to lay down a general rule. The issues may be healed on the first clear evidence of the formation of abscess; otherwise, if they occasion little or no inconvenience, they may be kept open for one or two years. The recumbent position should not be abandoned in less than six or seven months, even when the disease is in its earliest stage; and, in the great majority of cases, the period should be extended to a year, and sometimes to a year and a half.

"In the first instance, the surgeon usually finds it difficult to persuade the patient to continue this part of the treatment for a sufficient length of time after the removal of the more urgent symptoms. Afterwards, however, he has to encounter a difficulty of an opposite kind. This happens especially among young females; who often become at last so habituated to their couch, and the peculiar mode of life connected with it, that they can scarcely be persuaded to make the necessary effort to sit up and move about, even after every reason for not doing so has vanished. I know an instance of a lady, who, under these circumstances, has preserved the horizontal position for twelve years; and in whom nearly all the joints of the lower extremities, in which no actual disease ever existed, have, from mere want of exercise, become firmly anchylosed; so that it seems more than probable that nothing which can now be done will enable her to regain the use of the limbs, or even to sit up." 270.

The treatment of abscesses connected with a carious condition of the spine requires no peculiar observation. As a general rule, the patient should not venture to take exercise, nor even to quit the recumbent posture until the abscesses are healed. But Mr. Brodie mentions the case of a gentleman who had laboured for thirty-two years under well-marked symptoms of caries of the spine:—angular projection backwards, opposite the middle dorsal vertebræ, and sinuses communicating with them in a state of caries. This gentleman, with all this extent of disease, had been able to take violent exercise in hunting and in shooting, and had suffered from no material inconvenience, excepting the loss of the use of his lower limbs for a period of three months; this he regained after the application of blisters to the back.

This concludes the chapter upon caries of the spine—one very valuable, from the practical hints which it contains, to the young surgeon and the old. We proceed to another subject.

### On Tumours and Loose Cartilages in the Cavities of Joints.

Mr. Brodie has not much to offer on the subject of loose cartilages in the joints, the complaint having been so frequently described by writers.

In the majority of cases which he has witnessed, no symptoms of inflammation have preceded their appearance. He therefore thinks it probable that in some instances they are generated, like other tumors, in consequence of some peculiar morbid action. They appear to be situated originally, either on the external surface, or in the substance, of the synovial membrane; since, before they have become detached, a thin layer of the latter may be traced to be reflected over them.

"My own experience is much in favour of the removal of these loose cartilages by an incision of the joint, provided that this be done in a cautious and prudent manner. The patient should be kept in a state of the most perfect quietude for two or three days preceding, and for several days after, the operation. The cartilage having been well fixed, the different parts over it should be slowly and separately divided until it is exposed. The wound of the synovial membrane may be dilated by means of a probe-pointed bistoury, until it is of sufficient size to allow of the cartilage being extracted with a tenaculum; and the cut edges of the skin should be instantly replaced in contact with each other, and secured by means of adhesive plaster." 272.

Mr. Brodie has seen two cases in which the loose cartilages were of a different nature from those above alluded to. It occasionally happens that a bony ridge is formed, like a small exostosis, round the cartilages of the joint. In the two cases to which Mr. Brodie refers, this preternatural growth of bone had taken place, and portions of it had been broken off and lay loose in the cavity of the articulation.

*Small Pendulous Excrescences in the Knee-Joint.*

These spring from the synovial membrane, are numerous, pendulous, have a smooth external surface, and resemble in appearance the appendices epiploicæ of the great intestine, though they do not, like them, contain adipose substance. Mr. Brodie has seen three preparations of this description. With the history of one he is unacquained—there is reason to believe that long-continued inflammation of the synovial membrane was the cause of the excrescences in another—and in the third, they were accompanied with the collection of a considerable quantity of whey-like fluid in the joint.

*Other Tumors in the Joints.*

1. In a case of the late Mr. Ewbank's, at St. George's Hospital, a tumor existed within the knee-joint, situated on the edge of the patella and over the external condyle of the femur. It appeared like a loose cartilage, of about the size and form of an almond, and it produced such symptoms as loose cartilage occasions. Mr. Ewbank made a careful incision on the tumor, which proved to be of a gristly structure, and to be attached by one extremity to the synovial membrane, near the edge of the patella. This attachment was cut through, and the tumor was removed. The wound healed, and the patient experienced much relief, but six weeks afterwards a tumor was discovered in the knee of smaller size than that which had been removed, but occupying the same situation. This tumor could be pressed into the joint with the fingers, but did not slip into it in walking. When the patient left the hospital, he did not suffer any inconvenience from it.

2. A young man consulted Mr. Brodie on account of what seemed to be loose cartilage of the right knee. In certain motions of the joint a tumor presented itself on the inside of the patella, which appeared to be a loose cartilage of large dimensions. The symptoms supported this suspicion. He was desirous of an operation. This was cautiously performed by Mr. Brodie. The tumor was found to be of fleshy structure, and to be connected with the synovial membrane, below the patella, by a broad adhesion. Mr. Brodie divided it and removed the tumor. All subsequent precautions were adopted, but violent inflammation supervened, and required very active treatment. At the end of two months, the knee was neither swollen nor painful, but was still incapable of perfect flexion and extension.

"On examining more accurately the tumor which had been removed in this case, it was found to be about two inches and a half in length, and one inch and a half in breadth, and somewhat less than half an inch in thickness in the thickest part; convex on one surface and somewhat flattened on the other. It was of a firm fleshy structure. The general appearance of it a good deal resembled that of the coagulum which is found in the sac of aneurism; but it was not laminated; it had a smooth membranous surface; and it was manifestly organized, as vessels might be distinctly traced ramifying through its substance."

Mr. Brodie confesses that an exact acquaintance with the nature of the tumor in the two preceding cases, would probably have made him pause before he ventured to remove them by an operation. Yet, even with the narrative of those cases placed before us, it is difficult to ascertain the distinctive characters of tumors of this nature. Perhaps, a careful examination might enable us to determine that the tumor does not present the same degree of hardness as cartilage. It is fortunate that the latter is so much more frequent than the former as to render the chance of a mistake inconsiderable.

### Of Malignant Diseases of the Joints.

Mr. Brodie observes that the bones are well known to be liable to those morbid growths and alterations of structure, which are usually denominated malignant diseases.

"In the cases which have fallen under my observation, carcinoma of the bones has never occurred as a primary disease, but has always been preceded by carcinoma of the breast or some other glandular organ. The existence of the disease in the bones has been indicated by pains, sometimes slight, at other times most severe, resembling those of deep-seated rheumatism, but not yielding to the use of the remedies by which rheumatic pains are usually influenced. In these cases, the bones themselves become unnaturally brittle, and are so easily broken, that I have more than once known a fracture of the femur to be produced by the patient accidentally turning herself in bed; and, in one instance, a fracture of the clavicle took place on the patient making some slight effort in raising her arm." 280.

Mr. Brodie relates two cases of cancer of the bones. In the first, the head of the femur was affected, and the symptoms in some degree resembled those produced by disease of the hip-joint. The patient had undergone the operation for the removal of a scirrhous breast, and the disease had reappeared in the cicatrix. The femur was fractured at the seat of the disease by a very slight injury immediately before or after death. The whole of the superior extremity of the thigh-bone was softer and more brittle than natural, a change which was most distinct in that part of the head contiguous to the cartilage. On making a section of the head and neck of the bone, the earthy matter was found to be very deficient, and a cartilaginous or gristly substance was seen blended with the bony structure. In several places there were spots of increased vascularity with a deposition of some cheesy matter in the centre.

In the second case, the alteration attacked the spine. The patient had scirrhus of the breast, and was suddenly seized with paralysis of the whole lower part of the body. On dissection, several of the dorsal vertebræ were converted into a structure closely resembling that of the femur in the last case. The whole of the lower portion of the theca vertebralis was filled with a serous fluid.

Mr. Brodie observes that the bones are much more liable to be affected with fungus hæmatodes than with scirrhus, and that the former frequently occurs in them as a primary disease. Mr. Brodie has seen several cases in which a tumor of this description had its origin in one of the bones of a joint. It is obvious that such in its progress affecting contiguous parts must gradually render the joint useless, and end in its destruction.

"In these cases the patient first complains of a slight degree of pain in the affected part, which is somewhat aggravated by exercise. Some time afterwards the bone is observed to be slightly enlarged. As the tumour increases, it is found to be elastic in some parts, hard in others. For a considerable time it does not interfere with the functions of the joint; which, however, afterwards becomes limited in its motions, and, ultimately, completely fixed in one position. I have never known but one case in which the patient did not submit to amputation before the disease had reached its most advanced stage; and here the skin became ulcerated, and a large ill-conditioned sore was the consequence." 285.

Amputation is the only remedy, and those best acquainted with the results of operations for fungus hæmatodes will most readily appreciate the slender chances of success from even this cruel expedient. Mr. Brodie relates two cases of fungus hæmatodes, in which amputation was performed. In one the lower part of the femur was affected; in the other the head of the tibia was concerned. It is four years since the operation was performed for the first patient, and no return of the malady has taken place. Mr. Brodie does not mention the termination of the second case, but we understand that the patient died with pulmonary symptoms in less than a year after amputation.

### OF SOME OTHER DISEASES OF THE JOINTS.

Such is the heading of the ninth chapter of the work. The subjects included in the general designation are the following:—exfoliation of the epiphysis of a bone, as a consequence of acute inflammation—chronic inflammation of the epiphysis, sometimes terminating in abscess in the bone contiguous to the joint—absorption of the cartilages by a process which apparently differs from inflammation—anomalous pains in hysterical females referred to the joints or their vicinity—unusual liability to dislocations of the joints—osteo-sarcoma—a peculiar deposition between the dura mater of the spinal marrow and the bodies of the vertebræ—gouty alteration of a joint—acute ulceration of the cartilages—differences in the length of the two lower limbs. On these various affections Mr. Brodie offers in succession some remarks.

1. *Exfoliation of an Epiphysis from Acute Inflammation.*

Acute inflammation of the shaft of a bone and of its periosteum is usually arrested at the epiphysis. But some instances occur in which the epiphysis itself is attacked, and more or less extensive exfoliation is the consequence. Sometimes nearly the whole of the epiphysis is deprived of its vitality; at other times only one small portion of it, or several are affected. In some of these cases, the exact nature of the disease is sufficiently obvious; but in others, where the exfoliations are of a very small size, it is difficult or impossible to form an exact diagnosis. This is, however, of the less importance, as, under all circumstances, such a disease must terminate in the complete destruction of the joint; so that there is no remedy but amputation.

2. *Chronic Enlargement and Abscess of the Epiphysis.*

Chronic enlargement of the epiphysis from inflammation is not an unfrequent occurrence. It is liable to be mistaken for disease of the joint itself, especially as inflammation of the synovial membrane sometimes ensues as a secondary consequence. The patient may derive benefit from the treatment which is applicable to nodes in other portions of the bones.

Abscess in the centre of the bone contiguous to the joint is another result of chronic inflammation of the epiphysis. This produces excessive suffering, and has occasioned, perhaps frequently, amputation of the limb. Mr. Brodie has advocated the use of the trephine, pointing out the symptoms characteristic of the disease, and the value of the remedy alluded to, by a series of interesting cases. The description of this complaint was originally offered to the public by Mr. Brodie in the 17th volume of the Medico-Chirurgical Transactions. It was fully noticed in the 35th Number of this Journal, that for January, 1833. We must refer our readers to the number in question for a full account of the important observations of Mr. Brodie. We therefore pass on.

3. *Absorption of the Cartilage, independently of Inflammation.*

It is sometimes observed that the bone becomes partially denuded of cartilage, without any marks of inflammation. There is no erosion of the bony surface itself, and the remaining cartilage retains its natural adhesion to it. The patient does not complain of pain in the joint, nor does suppuration follow. These changes are observed more frequently in the bodies of elderly persons; and they are sometimes discovered after death, where their existence had not been suspected during the patient's lifetime. At other times, they produce in the motions of the limb a grating, corresponding to, but less distinct than, the grating which is perceptible after a fracture.

4. *Pains in the Joints in Hysterical Females.*

Instances are not uncommon of pains referred to the large articulations in hysterical females. These cases give rise to much anxiety, yet ultimately bad consequences seldom ensue.

"At first there is pain referred to the hip or knee, or some other joint, without any evident tumefaction; the pain soon becomes very severe, and, by degrees a puffy swelling takes place, in consequence of some degree of serous effusion into the cells of the cellular texture. The swelling is diffused, and, in most instances, trifling; but it varies in degree: and I have known, where the pain has been referred to the hip, the whole of the limb to be visibly enlarged from the crista of the ilium to the knee. There is always exceeding tenderness; connected with which, however, we may observe this remarkable circumstance, that gently touching or pinching the integuments, in such a way as that the pressure cannot affect the deep-seated parts, will often be productive of much more pain than the handling of the limb in a more rude and careless manner. In one instance, where there was this nervous affection of the knee, immediately below the joint there was an actual loss of the natural sensibility; the numbness occupying the space of about two or three inches in the middle of the leg. Persons who labour under this disease are generally liable to other hysterical complaints; and, in all cases, the symptoms appear to be kept up and aggravated by being made the subject of constant attention and anxiety." 303.

No general rule can be laid down, says Mr. Brodie, for the treatment of cases of this description. In fact, it may be broadly stated, that the measures applicable to the relief of the hysterical condition, are those which are required for this. To make this remark, is to state that the individual case must be studied. In one hysteric female, the menstruation is profuse—in another it is absent or defective; in one the bowels are extremely torpid—in another they are regular; in one the nervous system is peculiarly excitable—in another it is sluggish; in one the circulation is languid—in another the blood-vessels are loaded—in all there are irregular determinations of blood. The practical surgeon or physician must have witnessed these various conditions in females who are subject to the lengthened and the varied train of hysterical disorders. The management of the individual case requires and displays the judgement and resources of the medical attendant.

Some local applications appear to Mr. Brodie to be serviceable. When the pain is most severe, the patient occasionally derives benefit from the use of the following embrocation, applied tepid.

℞. Spiritus rosmarini, ℥iss.

Mist. camphoræ, ℥viss.

M. fiat embrocatio.

Or, a liniment of camphor and belladonna has seemed to be productive of advantage.

℞. Liniment. camph. comp. ℥iv.

Extracti belladonnæ, ʒij.

M. fiat linimentum.

Mr. Brodie observes that medical treatment is subservient, after all, to moral management. The patient's attention must be diverted from her complaint to other and engrossing objects; she should mix in society; take exercise out of doors, especially on horseback; rise early; and assume in all respects the habits of a healthy person.

"In general, it is not difficult to distinguish the cases which I have just described from those of more serious disease. Careless surgeons, however, frequently fail in their diagnosis; and even surgeons of experience do so in some instances. I do not hesitate to say, that a large proportion of young ladies, who have heretofore been supposed to labour under disease of the hip-joint, and the great majority of those who have been treated as suffering from caries of the spine, have, in reality, been affected with these local hysterical symptoms, and nothing more. Except where there is a question concerning life and death, no error in surgical practice can be more dangerous than this; as it may lead to a patient being confined to her couch, almost in solitude, for months, or even for years, who ought to be taking exercise, and breathing the fresh air, and partaking of the amusements, and enjoying the society of those of her own age." 305.

Perhaps there are few affections in which mistakes are more frequently committed by the generality of surgeons than in this. Yet its frequency in one form or another renders it a matter of the utmost consequence, both to the surgeon and the patient, that the former should be well acquainted with its nature. We could almost have desired that Mr. Brodie had entered more fully on its history, and had dwelt at greater length upon its symptoms. He might usefully have drawn from his large store of facts and of experience, more particular descriptions and more copious observations for the benefit of the mass of the profession. As it is, we would recommend an attentive study of the brief remarks which Mr. Brodie has offered.

5. *Unusual Liability to Dislocations of the Joints.*

A gentleman twenty-three or twenty-four years of age, consulted Mr. Brodie in the year 1820. He had met with the accident of dislocating the patella four times in the right, and once in the left knee. The right shoulder had been twice completely dislocated, and once there had been a subluxation of the same joint. The joint of the left thumb, with the *os trapezium*, had been dislocated several times. In every instance the dislocation had been reduced with the greatest facility, and generally without surgical assistance. The gentleman was in good health, and no peculiarity could be detected in the form and structure of his joints. His muscles were strong, and he was capable of considerable muscular exertion; he was accustomed to a good deal of walking exercise, but had not been particularly exposed to the ordinary mechanical causes of dislocation.

6. *Osteo-Sarcoma in the neighbourhood of Joints.*

Mr. Brodie relates one case, and alludes to three others, in which this disease fell beneath his observation. The first case was that of a lady. The part affected was the knee, and the tumor attained such a size that amputation was performed by Mr. Thomas. The tumor occupied the upper part of the knee, beginning at the edge of the cartilaginous surface, and extending about three or four inches up the lower part of the thigh. It was interposed between the muscles and the bone of the thigh, so that the former were seen expanded over it. It was of a greyish-white colour; composed of fibres of a gristly semi-transparent substance, with osseous matter intermixed with it, and about two inches in thickness on each side of the femur. At the upper part it was seen distinctly originating in the periosteum; at the lower part the periosteum could not be traced, and the structure of the bone was continued into that of the tumor. The cartilages and ligaments of the joint were free from disease.

Mr. Brodie observes that there can be no question of the original disease being osteo-sarcoma, and of its origin in the periosteum of the femur. In the other three cases alluded to the disease was connected with the hip. The patient appeared to labour under an enormous tumor of this joint. Mr. Brodie was only able to examine one case by dissection. The hip itself was free from disease, and the enlargement was formed by an osteo-sarcomatous growth from the periosteum of the upper extremity of the femur.

7. *Peculiar Deposition on the Spinal Dura Mater.*

Mr. Brodie appears to have seen but one case of this description, and he relates it, not only for the purpose of displaying the peculiar malady, but also with the view of illustrating the observation previously made—that disease affecting the cervical portion of the spinal chord produces paralysis of the upper extremities in the first instance, and of the lower extremities afterwards.

*Case.* A young man, after leading a very irregular life, and after much exposure to damp and cold was seized, in January, 1829, with a violent pain in the neck, succeeded by considerable swelling. This was chiefly situated on the right side, extending from the head to the shoulder. Although the patient neglected himself the pain and swelling in a great degree subsided. In the beginning of the following April, the upper extremity of the right side became affected with paralysis. Afterwards the opposite limb became, to a great extent, paralytic also. He remained in this state till June, when he came under the care of Mr. Brodie.

"At this time he complained of some degree of pain in the back of the head and neck; and he found it difficult to move the head from one side to the other. An enlargement and induration of the soft parts of the neck was still perceptible in the situation of the original swelling. There was complete paralysis of the muscles of the right arm, forearm, and hand: those of the opposite limb were also paralytic, but some of them were still capable of acting feebly, so that he could take hold of the right hand with the left, and move it from one position to another. The muscles of the lower limbs were feeble, but were capable, nevertheless of supporting the body in the erect posture.

The bowels were very torpid, and the evacuation of a dark colour, a good deal resembling tar in appearance.

The urine was slightly alkaline, but voided without difficulty." 310.

Leeches, a seton, mercury were employed without effect. The lower limbs became paralytic, and, in the latter end of June, the patient died, having previously been comatose.

On dissection, the ventricles of the brain were found to contain about two ounces of watery fluid, and the brain itself was unusually soft. The cervical portion of the spinal chord was also softer than natural.

"A quantity of soft solid substance, of a grey colour, apparently lymph, which had become organised, was found situated between the dura mater and the bodies of the vertebræ, occupying the whole of the anterior and some of the posterior part of the vertebral canal, and extending from the occiput downwards, as low as the fourth cervical vertebra.

A substance similar to that which was found on the inside of the vertebral canal was also found lying on the fore part of the bodies of the cervical vertebræ, extending over the oblique and transverse processes, and communicating with the internal mass by processes extending through the spaces in which the nerves are situated, and surrounding the nerves themselves. The external mass was much larger than the internal, being not only thicker, but extending lower down in the neck. In some parts it was not less than an inch in thickness: in other parts thinner, and, altogether, it was of a very irregular shape." 311.

8. *Gouty Alteration of the Joints.*

These are remarkable. The cartilages are absorbed; the exposed surfaces of bone are partially or entirely encrusted with a white earthy matter, apparently lithate of soda; and sometimes they have the appearance of being worn into grooves. In some cases, repeated and long-continued attacks of gout occasion complete anchylosis.

### 9. *Acute Ulceration of the Cartilages.*

Mr. Mayo has published some cases of this description, and Mr. Brodie has also seen some. Our readers will perceive an account of them in the article contained in the last number of this Journal.

### 10. *Difference in the Length of the Lower Limbs.*

"It occasionally happens that the two lower extremities are not of precisely the same length; and this may be the result of original formation, the femur and tibia of one side being respectively longer than those of the other side. If the whole of this difference amounts, as it sometimes does, to an inch, or an inch and a half, the individual is observed to limp in walking, and the great trochanter belonging to the longer limb is higher and more prominent than that of the other; and this might lead a superficial observer to mistake the case for one of diseased hip." 313.

In some instances disease is a cause of difference in the length of the two limbs, a diseased bone not keeping pace, in growth, with a sound one. But Mr. Brodie relates a case in which, after necrosis of one femur, the thigh was found by careful and repeated measurement to be an inch and a quarter longer than its fellow. These circumstances should be remembered, as a patient might erroneously be supposed to be labouring under disease of the hip-joint, in consequence of the alteration of the figure of the limb.

We now arrive at the tenth and last chapter. It is dedicated to the subject of inflammation of the bursæ mucosæ.

### On Inflammation of the Bursæ Mucosæ.

Under the general designation of bursæ, Mr. Brodie includes, for the sake of convenience, the membranes forming the sheaths of the tendons.

"Inflammation of the bursæ mucosæ is marked by nearly the same characters, and (allowance being made for the difference of the parts with which they are connected) produces nearly the same results as inflammation of the synovial membranes of the joints. In the greater number of instances, it occasions an increased secretion of synovia. In other cases the bursa is distended by a somewhat turbid serum, with portions of coagulated lymph floating in it. Occasionally it terminates in the formation of abscess. Sometimes the membrane of the bursa becomes thickened, and converted into a gristly substance. I have seen it at least half an inch in thickness, with a small cellular cavity in the centre containing synovia. At other times, although the inflammation has continued for a very long period, the membrane of the bursa retains nearly its original structure." 317.

Inflammation of the bursæ may be the consequence of pressure or of other local injury—of abuse of mercury, of rheumatism, or of some other constitutional affection; in these latter cases it is frequently combined with inflammation of the synovial membranes of the joints. Sometimes it assumes an acute, more frequently a chronic form.

The inflamed bursa forms a tumor, more or less distinct, according to its situation ; more or less painful, according to the character of the inflammation. If superficial fluctuation is perceptible at first, and if, under these circumstances, the inflammation is considerable, the surrounding parts are implicated, and the skin is red. When the disease has existed for any length of time, the membrane has generally become thickened, sometimes to the extent of making the bursa resemble a solid tumor.

When the inflammation is of long standing, it is not unusual to find floating in the fluid of the bursa a number of loose bodies, of a flattened oval form, of a light brown colour, with smooth surfaces, resembling small melon-seeds in appearance. There seems to be no doubt that these loose bodies have their origin in the coagulated lymph which was effused in the early stage of the disease, and Mr. Brodie has traced the steps of their gradual formation, from irregular masses, of no determined shape, to smaller portions, and ultimately to the flat oval bodies, described. Motion and pressure are the agents of change.

When suppuration takes place in a bursa, the abscess sometimes makes its way directly to the surface of the skin, and bursts externally. But Mr. Brodie suspects that, in other cases, the matter first escapes into the surrounding cellular texture, and the case is then liable to be confounded with those in which the abscess originates in this tissue. Mr. Brodie is induced to entertain this suspicion, from the consideration of the following circumstances.

"There is no bursa more liable to be inflamed than that between the patella and the skin ; and inflammation of it not unfrequently terminates in suppuration, as I have ascertained to be the case, both by the discharge of pus, when the tumour has been punctured, and by dissection after death. It is very common to find a large abscess on the anterior part of the knee, which the patient describes as having commenced over the centre of the patella in the situation of this bursa. The abscess has a somewhat peculiar character. It raises the skin from the patella, so that the latter cannot be felt, and from this point as from a centre, it extends itself between the skin and the fascia, equally in every direction, covering the whole of the anterior part of the knee. A superficial observer, judging from the general form of the tumour, and the fluctuation of fluid, without noticing the greater redness of the skin, and the circumstance of the fluid being over, instead of under, the patella, might mistake the case for one of inflammation of the synovial membrane of the joint itself. Such an abscess must be supposed to commence either in the bursa above mentioned, or in the cellular texture. The original situation of the disease corresponds to that of the bursa: there appears to be no reason why an abscess of the cellular texture should occur in this precise spot, more frequently than elsewhere; and hence, it is reasonable to conclude, that the bursa is the part in which the abscess begins. It is not improbable that many other abscesses of the extremities may have a similar origin. The tumour which occurs in the inside of the ball of the great toe, and which is one of those to which the name of bunion has been applied, occasionally suppurates ; and I have found, on dissection, that this is formed by an inflammation of the bursa, which is here situated." 320.

It frequently happens that, after the inflammation has entirely subsided, a disposition to the secretion of a preternatural quantity of fluid still remains, and dropsy of the bursa is the consequence. Such tumors are very frequent in the neighbourhood of the wrist, and are sometimes confounded with ganglions. The enlarged bursa on the anterior part of the wrist has somewhat peculiar characters; it is bound down in the centre by the strong annular ligament, which binds down the flexor tendons; and it is prominent above and below, where the superjacent parts afford a smaller degree of resistance. Pressure made on the upper part of the tumor causes the fluid to pass altogether into the palm of the hand, and, in like manner, pressure on the lower part of it causes it to ascend into the fore-arm.

These are all the remarks which Mr. Brodie offers on the history and symptoms of inflammation of the bursæ. He next proceeds to the consideration of the treatment.

In the first instance, this should be guided by the principles which usually regulate our management of inflammatory affections. Leeches, lotions, blisters will be requisite, and colchicum, or such constitutional remedies as appear to be adapted to the individual case, will readily occur to the mind of the experienced surgeon.

When dropsy of the bursa has occurred, the application of a splint or bandages to obviate motion, and of blisters, kept open by the savine cerate, are the best means of promoting the absorption of the fluid. But when the loose bodies, resembling melon-seeds, are contained in the bursal cavity, it is necessary to evacuate them by a puncture. Having noticed that the supervention of suppuration, after this trifling operation, was usually productive of a permanent cure, Mr. Brodie has sometimes been induced to procure the suppurative process artificially, by the introduction of a seton or tent into the wound, or by opening the bursa freely, and dressing the cavity with lint. Even where the bursa forms the sheath of one or more tendons, this method may be employed with safety; provided that the bursa has no communication with the cavity of the neighbouring joint.

"We must, however, proceed with caution where the bursa is dilated to a considerable size. Inflammation and suppuration of a large bursa sometimes disturbs the constitution in so great a degree, that it may be doubtful whether it would be prudent, in this instance, to do more than simply puncture the tumour, keeping the patient in a state of perfect quietude afterwards. A large swelling, formed by a cyst distended with serum only, or with serum and masses of coagulated lymph floating in it, occasionally is met with over the inferior angle of the scapula; originating either in the large bursa mucosa, which is interposed at this part between the *scapula* and the *latissimus dorsi* muscle, or otherwise in one of the bursæ of the shoulder, protruding from underneath the muscles by which that joint is surrounded. I had on opportunity of seeing a tumour of this description, which had attained a magnitude not much less than that of a man's head. I understood that the cyst was afterwards punctured, and a seton passed through its cavity, and that so much disturbance of the general system ensued, as to occasion death. I have seen another case, in which death took place in a short time after such a tumour was punctured: but here the patient was otherwise in bad health, and that strict attention was not paid to his being kept in a state of quietude after the operation, which the circumstances seem to have required. I shall give an account of a more fortunate case of the same kind hereafter." 323.

Where the coats of the bursa have become much thickened, there appears to be no remedy but its removal by an operation. This is applicable to superficial bursæ only; for where they envelop the tendons, it would be impracticable, and where they communicate with the cavity of a joint it would be improper. Mr. Brodie has only found occasion to perform it on the bursa which intervenes between the patella and the skin. The readiness with which new bursæ, or organs which perform their office, are generated in situations in which they are required, would dissipate any uneasiness, were it entertained, respecting the removal of a diseased one.

Mr. Brodie relates five cases, illustrative of the history and treatment of diseases of the bursæ. At these we shall cast a hurried glance.

In the first case, the bursa over the patella was enlarged and contained fluid; but its coats were very little thickened. Ordinary means having failed, Mr. Brodie punctured the tumor, and irritated its interior with the blunt end of a probe. Suppuration ensued, and a cure was effected.

In the second case, the bursa over the inferior angle of the scapula was enlarged to the size of a cocoa-nut. Mr. Brodie punctured it with an abscess-lancet, and discharged about a pint of turbid serum, with some irregularly-shaped masses of coagulated lymph. Suppuration ensued, but, after the expiration of three months, it had ceased, the wound cicatrized, and no remains of the tumor were perceptible.

In the third case, the bursa enveloping the extensor tendons of the wrist was enlarged to about the size of a double walnut, and was filled with fluid. This was discharged by a puncture, but re-accumulated, and Mr. R. Keate made a longitudinal incision in the skin over the tumor, and dissected out as much as possible of the bursa, leaving only that part of it which enveloped the tendons. Suppuration ensued. In a few weeks after the wound had healed, the tumor re-appeared with the same characters as before, but of only half its former size. In that condition it has since remained.

In the fourth case, Mr. Brodie dissected out a thickened bursa from between the patella and the skin. The parietes were more than half an inch in thickness. The patient was cured.

In the fifth case, a tumor, having all the characters of an enlarged bursa, appeared in the site of one which had been removed from between the patella and the skin. Mr. Brodie was compelled to cut into it, and to dress the wound with lint, which measures were productive of a cure.

The last fourteen pages of the work are occupied with a refutation of the arguments of Mr. Key, on the mode in which the cartilages of joints are ulcerated. We have dwelt so much upon this subject, that it does not seem necessary to enter on it again.

Here, therefore, we part from Mr. Brodie. To express our real feelings on the merits of his work might be construed into adulation. We observed at the commencement of our analysis, for a review it has not been, that we held up the book to the medical world as a valuable model. We have placed its substance before our readers—have diffused its accumulated facts—displayed its spirit of philosophical induction; and we venture to indulge the hope, that those of our elder brethren who are not familiar with it will become so, and that every young surgeon will consider it an indispensable ingredient in his library, meagre as his circumstances may compel it to be. It is not merely that the mass of information is great, but the admiring reader observes with gratification and instruction, the application of an original mind to a complicated subject, and the philosophical truth of the inferences elicited by a strict adherence to inductive reasoning. The theorist will seal the merits of the author, by hinting that he displays no graces of fancy, no charms of imagination. Again we recommend every surgeon and physician to purchase this edition.

